# The OPT2MISE Study – A Review of the Major Findings and Clinical Implications

**DOI:** 10.17925/EE.2015.11.02.70

**Published:** 2015-08-19

**Authors:** Yves Reznik, Michael Joubert

**Affiliations:** Endocrinology and Diabetes Department, CHU Côte de Nacre, Caen Cedex, France

**Keywords:** External insulin pump, CSII, type 2 diabetes, intensified insulin therapy, anti-GAD antibodies, C-peptide

## Abstract

Many patients with type 2 diabetes struggle to achieve adequate glucose control despite escalation of therapy including complex insulin regimens with multiple daily injections (MDIs). Previous randomised controlled trials failed to show a significant improvement in glycaemic control with pump therapy over multiple injections. The OPT2MISE study enrolled 495 adult patients with poorly controlled type 2 diabetes despite an intensified insulin regimen using rapid and slow-acting insulin analogues. After a 2-month run-in period, patients were randomised to switch to pump therapy or to maintain their MDI regimen. After 6 months, patients with pump therapy achieved a better glycaemic control than those who used multiple injections (glycated haemoglobin [HbA_1c_] difference of −0.7 %), and twice as many patients reached the target range of 8 % or less in the pump-therapy group compared with the injection group. Patients using pump therapy had a 20 % reduction of their total daily insulin dose. A moderate weight gain was observed with both treatments, and no severe hypoglycaemia nor ketoacidosis occurred in the pump therapy group. Pump therapy may now be considered as a valuable option in type 2 diabetes patients who fail to respond to an intensified insulin regimen.

In type 2 diabetes (T2D), the addition of basal insulin is an option when oral therapy does not maintain acceptable glycaemic control. For the 50-60 % of patients who become refractory to basal insulin therapy alone^1,2^ treatment intensification requires the addition of prandial insulin to target control of post-prandial hyperglycaemias. The resulting regimen of multiple injections of rapid-acting insulin with basal insulin (MDI) achieves target glycaemia in only 70 % of patients, the remaining corresponding to MDI failure.^3^ As an alternative, glucagon-like peptide-1 (GLP-l)-receptor agonists should be combined with basal insulin, this association thus providing equal efficacy compared with the combined basal bolus strategy and giving other advantages, such as weight loss and reduction of the hypoglycaemia rate.^4–7^ The use of external pumps in patients with T2D is a recent practice compared with that in type 1 diabetes. In only a few countries, including France, Sweden and Israel, is continuous subcutaneous insulin infusion (CSII) using an external pump an alternative to multiple injection therapy in T2D and can also be reimbursed by the relevant health authorities. Before 2014, clinical trials evaluating pump therapy in T2D were scarce^8–11^ and retrospective reports on its use in clinical practice were rather limited.^12–16^ In 2014, the large multicentre, controlled trial, OPT2MISE, which compared pump therapy with multiple injections in insulin-treated patients with T2D demonstrated the superiority of CSII over multiple injections and better defined which patients may be a candidate for pump therapy.^17^ In this review, the state of the art concerning the use of CSII in T2D before OPT2MISE and the major findings and lessons from the OPT2MISE trial will be overviewed.

## Pump Therapy in Type 2 Diabetes before OPT2MISE

The first case reports on CSII utilisation in T2D were published in the 1980s showing that pump therapy was an effective tool in patients with extreme insulin resistance and poor glycaemic control. In these studies, insulin was administered as a transient intravenous insulin infusion, which lowered the mean glucose level and reduced insulin requirements by 40 %.^18,19^ A sequential utilisation of intravenous insulin infusion for 4 weeks followed by CSII for 1 year was undertaken by Pouwels et al. in eight obese T2D patients with diabetes with poorly controlled diabetes despite high insulin requirement (~2 U/kg/day). This sequential treatment allowed them to reach near normal glucose levels within a few weeks, with a reduction of glycated haemoglobin (HbA_1c_) from 12 % to 8.9 % and a 35 % reduction in insulin requirement. Insulin sensitivity was dramatically improved by intravenous insulin. One year after the switch to CSII, mean HbA_1c_ was maintained below 8.5 %.^20^ Another pilot study in Israel performed in 10 patients with poor glycaemic control also demonstrated a dramatic improvement in glucose control together with a 20 % reduction in insulin requirement after 40 weeks of CSII utilisation, suggesting that insulin sensitivity or its bioavailability may be enhanced by CSII.^21^ Parkner et al. have demonstrated that switching from glargine to CSII at the same daily dose decreased by 10 % plasma glucose concentrations, increased insulin concentrations and gave a more flat plasma insulin profile, suggesting an enhanced absorption/bioavailability of the insulin administered with a pump device.^22^

**Table 1:** Randomised Controlled Studies Comparing CSII with MDI in Patients with Type 2 Diabetes

First Author/ Year (Reference)	Design	Number and Patient Characteristics (BMI, DD)	Anti-diabetic Treatment before CSII	Insulin Requirement before CSII (U/kg)	Baseline HbA_1c_ (%) CSII versus MDI	Final HbA_1c_ (%) CSII versus MDI	Weight gain (kg) CSII versus MDI	Type of Insulin Average Insulin Requirement	CGM data	Non-severe Hypoglycaemia Frequency
Raskin 2003^8^	Randomised parallel CSII versus MDI 24 weeks	n=127, BMI 32 DD: 12–14 years	Insulin+OHA 41 % Insulin only 59%	0.69 versus 0.75 U/kg/d	8.2 % versus 8 % 66 versus 64^¶^	7.6 % versus 7.5 % 60 versus 58^¶^ (NS)	+1.7 versus +0.8 (NS)	MDI/asp+NPH CSII/asp 0.6 versus 0.7 U/kg (NS)	NA	54 % versus 59 % (NS)
Herman 2005^9^	Randomised parallel CSII versus MDI 48 weeks	n=98, BMI 32 DD: 15–17 years	Insulin+OHA 43% Insulin only 57%	NA	8.4% versus 8.1% 68 versus 65^¶^	6.6 % versus 6.4 % 49 versus 46^¶^ (NS)	+2.1 versus +2.6 (NS)	MDI/lispro+glargine CSII/lispro 108 U/d	NA	81 % versus 90 % (NS)
Berthe 2007^10^	Randomised cross-over CSII versus MDI* 2x12 weeks	n=17, BMI 33.7 DD: 16.8 years	2 daily injections	1 U/kg/d	9 %	−1.3 versus −0.4 %* p<0.03	No change in both groups	CSII/lispro MDI/lispro+NPHx31 U/kg/d stable in both groups	Hyperglycated AUC CSII<MDI no difference in hyperglycated AUC	41 % versus 47 %
Wainstein 2005^11^	Randomised cross-over CSII versus MDI* 2x18 weeks	n=29, BMI 30–45 DD: NA	2-3 daily injections	>1 U/kg/d	10.2% and 10.3% 88 versus 89^¶^ (groups 1 and 2)*	−0.4 % versus +0.8 % (p<0.01)	−0.04 versus +0.09	CSII/lispro MDI/ regular+NPH metformin both groups −15 U/d versus +20 U/d	Hyperglycated AUC CSII<MDI	6 % versus 20 % (NS)
Reznik 2014^12^	Randomised parallel	n=331, BMI 33±7.2 DD: 15.1 ±8 years	Rapid and slow analogues ≥3 daily injection	0.7–1.8 U/kg/d	9±0.75 and 9±0.76	7.9±1.06 versus 8.6±1.24 (p<0.001)	+1.5 and +1.1 (P>0.1)	CSII/rapid analogue slow and rapid analogues metformin 70% both groups	24-hour AUC CSII<MDI	8.8 versus 5.1 mn/d

*Patients were randomised to begin with continuous subcutaneous insulin infusion (CSII) or multiple daily injections (MDIs) (groups 1 and 2, respectively);^¶^ mmol/mol. AUC = area under curve; BMI = body mass index; CGM = continuous glucose monitoring; DD = diabetes duration; HbA_1c_ = haemoglobin; NS = not significant; ОНА = oral hypoglycaemic agents; NPH = neutral protamin Hagedorn; NA = not available.

**Table 2:** Characteristics of the Patients Enrolled in the OPT2MISE Study

	CSII (n=168)	MDI (n=163)
Age (years)	55.5±9.70	56.4±9.50
Gender (men/women) (n, %)	94 (56.0 %)/74 (44.0 %)	86 (52.8%)/77 (47.2 %)
Duration of diabetes (years)	14.9±7.99	153±7.96
Body mass index (kg/m^2^)	33.5±7.50	33.2±6.99
HbA_1c_ (%)	9.0±0.75	9.0±0.76
Therapies		
Total daily long-acting insulin dose (units)	5.74±30.3	52.4±27.7
Total daily rapid-acting Insulin dose (units)	55.6±31.7	53.8±30.8
Insulin dose (units/kg per day)	1.1 ±0.4	1.1 ±0.4
Using metformin	341 (69.0 %)	341 (69.0 %)

*CSII = continuous subcutaneous insulin infusion; HbA_1c_ = glycated haemoglobin; MDI = multiple daily injections*.

Before OPT2MISE, few randomised controlled studies had compared the effectiveness of CSII versus MDI and had actually drawn contrasting conclusions (see *Table 1*).^8–11^ Two parallel-group studies were conducted in type 2 obese patients with diabetes treated with insulin therapy including at least one daily injection, with a mean HbA_1c_ between 8 % and 8.4 %. Treatment-intensification strategies compared CSII with a basal/bolus regimen during a 6 to 12 month period and showed a HbA_1c_ lowering of the same magnitude with both MDI and CSII treatments.^8,9^ By contrast, two cross-over studies have shown an advantage of CSII in comparison with MDI. In these studies, obese T2D with diabetes were randomly and successively assigned to CSII or MDI for periods of 12 and 18 weeks, respectively. Importantly, baseline HbA_1c_ was above 9 % despite high insulin requirements and multiple daily injections. In these studies, CSII was more effective than MDI for lowering HbA_1c_ with a between-group treatment difference of 1.2 % and 0.75 % respectively. Continuous glucose monitoring performed in both studies showed a significant reduction of glucose area under the curve with CSII in comparison with MDI.^10,11^ The conclusions that can be drawn from these studies are limited due to the small size of the sample populations, the lack of clear selection criteria for patient enrolment and the heterogeneity of insulin regimens used prior to and during the trials. Nevertheless, the discrepant conclusions of these studies may be explained by the differing patient’s profiles. In the two studies showing an advantage of CSII on MDI,^10,11^ baseline patient characteristics suggested more difficult to treat diabetes with higher insulin requirement, higher HbA_1c_ level and a higher number of insulin injections in comparison with the two studies showing similar efficacy of CSII and MDI.^8,9^

Pump therapy has also been successfully used in T2D patients with long-term follow-up in real-life conditions. A single centre longitudinal retrospective study in France in 102 poorly controlled T2D patients (HbA_1c_ 9.3 %) showed a −1.5 % HbA_1c_ drop after the switch from a MDI regimen to CSII with no change in insulin requirement. Such efficacy was maintained during a mean 5-year follow-up, suggesting the durability of CSII efficacy for glucose control. Interestingly, the subgroup of patients with a baseline HbA_1c_ below 8 % did not show a significant improvement in glucose control with CSII.^12^ Another French single centre study included 51 obese T2D with poor glucose control (HbA_1c_ 9.4 %) despite a regimen combining oral antidiabetic agents and basal insulin. After a twofold progressive increase in total insulin daily dose on CSII, HbA_1c_ dropped by −1.7 % and the benefit was maintained during a 7-year period of follow-up, indicating durability of CSII efficacy in the treatment of T2D.^13^ A third observational study from 31 French hospitals has reported results after a 2-year follow-up of 100 obese T2D patients previously treated by an intensified MDI regimen and switched to CSII. CSII was offered when HbA_1c_ was above 8 % and resulted in a −1.2 % drop at 1 year, which was maintained over 2 years of follow-up while insulin doses decreased by 25 % from baseline.^14^ Two other studies of shorter duration and with smaller cohorts of subjects found an improvement in glucose control in patients previously treated by various insulin regimens including a combination of rapid- and slow-acting insulin analogues. In these studies, baseline HbA_1c_ was ~8 % and dropped by −1.2 and −0.5 % respectively.^15,16^ These studies provide real-life data and although lacking a control arm, suggest the durability of CSII efficacy for a sustained glycaemic control.

Overall, the literature on CSII in T2D suggests that pump therapy should be a valuable option in selected patients who fail to respond to MDI therapy despite efforts to increase the insulin doses. Nevertheless, no clear demonstration of such a benefit was available until the publication of the OPT2MISE trial.

## Lessons from the OPT2MISE Trial

OPT2MISE was a prospective, randomised, controlled, parallel group study aiming to evaluate the comparative efficacy of MDI regimens in insulin-using patients with T2D, suboptimally controlled with advanced basal-bolus therapy.^17^ A total of 35 centres participated, including eight in Canada, 20 in Europe and Israel and three in the US. Patients were eligible to be enrolled in the study if they were 30–75 years old, had been diagnosed with T2D and were using insulin in a total daily dose (TDD) of 0.5–1.8 U/kg, not exceeding 220 U/day. Patients were required to have an HbA_1c_ above 8 % and below 12 % and to have been using an insulin regimen consisting of at least three injections per day of long- and rapid-acting insulin analogues for at least 3 months prior. Before randomisation, patients underwent a 2-month run-in phase during which insulin doses were increased using a standardised titration protocol with pre- and post-prandial glycaemic targets. Patients non-compliant to self-monitored blood glucose assessment during the run in phase were not allowed to participate to the study phase. This pre-study phase was designed to optimise glucose control with a basal–bolus insulin combination, which is the gold standard insulin regimen recommended by the American Diabetes Association and the European Association for the Study of Diabetes (ADA/EASD) guidelines for insulin intensification.^4^ Thereafter, patients whose HbA_1c_ levels remained above 8 % but below 12 %, who had performed at least 2.5 blood glucose self-assessments per day, and who had daily insulin requirements of 0.7–1.8 U/kg (maximum 220 U/day), were randomised in a 1:1 ratio to continue injection therapy or to receive pump therapy. The study phase had a 6-month duration and the primary endpoint was the between-group difference in change in mean HbA_1c_ from baseline. Blinded continuous glucose monitoring data were obtained using the Medtronic *i*Pro2 and the secondary endpoints included changes from baseline to 6 months in continuous glucose monitoring parameters including the time spent with hypoglycaemia and hyperglycaemia. The number of severe hypoglycaemic events and the episodes of ketoacidosis events were also recorded. Change in weight and insulin dose requirement were measured in both study groups between baseline conditions and the end of the study phase. Of the 590 patients who were assessed for eligibility, 472 entered the 2-month run-in phase, 141 were excluded at the end of the run-in phase and 331 were randomised to either CSII (n=168) or to continue using multiple daily injections (n=163). The patient’s characteristics are summarised in *Table 2*. Their mean HbA_1c_ was 9 %, mean total insulin daily dose 1.1 U/kg/day and 69 % of patients were on metformin.

**Figure 1:**
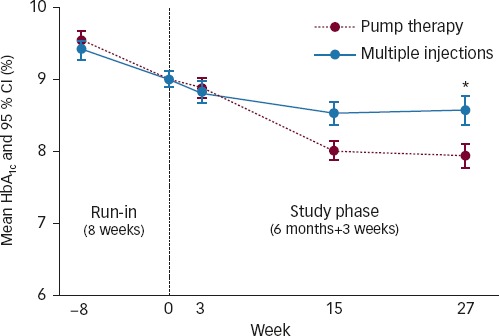
Glycated Haemoglobin Levels at Baseline, Randomisation, 1, 3 and 6 Months in Both Treatment Groups *p<0.001. HbA_1c_ = glycated haemoglobin; CI = confidence Interval. Source: Reznik Y, 2014.^17^

**Figure 2:**
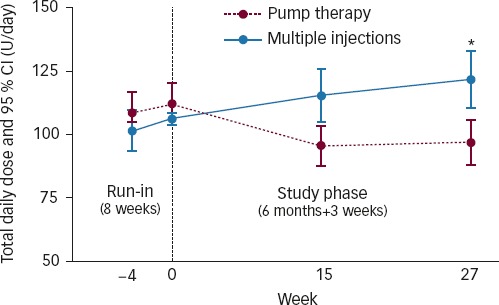
Daily Insulin Doses at Baseline, Randomisation, 1, 3 and 6 Months in Both Treatment Groups *p<0.001. CI = confidence interval. Source: Reznik Y, 2014.^17^

The main results of the study are summarised in *Table 1*. After they entered the study, patients on CSII had a rapid HbA_1c_ drop and after 3 and 6 months mean HbA_1c_ had decreased by 1.1 % compared with 0.4 % in MDI patients, with a between-group difference of −0.7 % (95 % confidence interval [CI] −0.9 to −0.4; p<0.001) favouring pump therapy (see *Figure 1*). Twice as many patients (95 % CI 1.5–2.5) reached the goal of HbA_1c_ below 8 % in the CSII group compared with the MDI group (55 % versus 28 %). Interestingly, patients with the lowest baseline HbA_1c_ (8–8.5 %) had few metabolic advantages with CSII compared with MDI patients (between-group HbA_1c_ difference of −0.3±0.85 %; p=0.105). Continuous glucose monitoring recordings showed a relative reduction in the duration of hyperglycaemic events with the CSII group (−168 mn per day; p<0.001) and in the area under curve for hyperglycaemia occurrence (p<0.01) in comparison with the MDI group. At the end of the study phase, the total daily insulin dose was 20 % lower in those on CSII compared with MDI (97±56 versus 122±68 U/d, respectively; p<0.001) (see *Figure 2*), such a reduction being at the expense of the bolus dose while basal insulin dose remained stable. No ketoacidosis occurred in neither the CSII nor the MDI group in terms of safety endpoints. One severe hypoglycaemia occurred in the MDI group and none in the CSII group. Continuous glucose monitoring analysis of hypoglycaemia events performed during a 6-day period at the end of the study phase showed a limited time spent in hypoglycaemia in both CSII and MDI groups without significant between groups difference (8.8 versus 5.1 mn/d; p=0.766). Thirty device-related non-severe adverse events occurred in CSII group compared with three in the MDI group. Weight gain was limited in both CSII and MDI groups without significant difference (+1.5±3.5 versus +1.1±3.6 kg; p=0.322). Treatment satisfaction was assessed by the DTSQ questionnaire and CSII was associated with significantly improved treatment satisfaction and reduction of hyperglycaemia perception in comparison with MDI. CSII was used in a simple way, most patients did not use the bolus calculator, and its use was not associated with the better metabolic efficacy of pump therapy.

## Possible Limitations on the Use of Pump Therapy in Type 2 Diabetes

**Figure 3:**
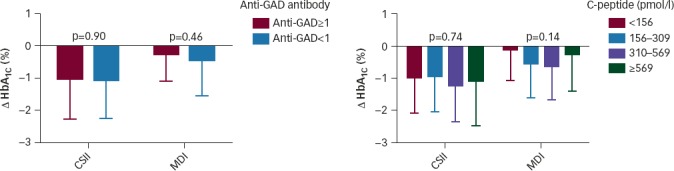
Glycated Haemoglobin Changes from Baseline in Patients from Both CSII and MDI Groups Stratified According to Anti-GAD Antibody Status and Quartile of C-peptide Level Anti-GAD = anti-glutamic acid decarboxylase; CSII = continuous subcutaneous insulin infusion; HbA_1c_= glycated haemoglobin; MDI = multiple daily injection. Source: Reznik Y, 2014.^25^

CSII is considered to be a complex therapy and its use involves a lot of cognitive and technical skills. Its convenience may therefore be questioned for its use in older patients with T2D. Interestingly, all patients enrolled in the OPT2MISE trial filled the Montreal Cognitive Assessment (MoCA) form, which has been validated for the detection of mild cognitive impairment. MoCA score was previously demonstrated to correlate with self-care autonomy in T2D pump users.^23^ Thirty-eight percent of patients in both OPT2MISE study groups had cognitive impairment detected by the MoCA score, but without any influence on CSII efficacy or safety. Pump therapy may therefore be used in the subset of patients likely to respond to CSII. Another matter has been raised by a panel of experts from the American Association of Clinical Endocrinologists (AACE) concerning residual beta cell function in T2D patients who are candidates for pump therapy. The recent guideline from the AACE stated that CSII may be suitable for selected T2D patients with suboptimal glucose control and a detectable C-peptide plasma concentration.^24^ Another recent guideline from the ADA/EASD stated that pump therapy may be an option in T2D patients with latent autoimmune diabetes of adulthood (LADA).^4^ The OPT2MISE trial provided an opportunity to evaluate the influence of C-peptide level and the presence of anti-glutamic acid decarboxylase (anti-GAD) antibodies detection in the OPT2MISE cohort. Changes in HbA_1c_ from baseline to 6 months were compared in patients categorised according to baseline anti-GAD levels (<1 or ≥1 U/ml), and to baseline C-peptide levels stratified into quartiles. There was no significant difference in HbA_1c_ change from baseline between subjects with positive versus negative anti-GAD antibodies, nor between subjects stratified according to C-peptide quartiles (see *Figure 3*). The benefits of pump treatment depended on neither anti-GAD antibody presence nor on C-peptide concentrations.^25^

## Intensification of Insulin Therapy – What Place for Pump Therapy?

Pump therapy may be an option in patients on a basal bolus regimen who do not reach glycaemic goals despite an active titration of insulin multiple injections. Although no direct comparison between pump insulin therapy and a combination regimen, which associates basal insulin plus GLP1-receptor agonist has yet been performed, such association may be considered as a realistic option before considering the initiation of pump therapy in patients on a MDI regimen.

## Summary

The OPT2MISE study clearly demonstrates for the first time that pump therapy used in a subset of obese insulin-resistant T2D patients with poor glycaemic control gives an advantage to optimised multiple injection regimen based on a greater decrease in HbA_1c_ and a reduction in the daily dose of insulin. Pump therapy may be considered as a safe option, and a simple use of the device does not preclude its efficacy and safety. Advantages of pump therapy may include improved convenience for patients, lessening of the cognitive burden associated with dose tracking and scheduling and decreasing insulin injection omissions. Pump therapy may be considered as a new treatment option in the armamentarium of T2D treatment intensification.

## References

[1] Nathan D. M., Buse J. B., Davidson M. B. (2009). Medical management of hyperglycaemia in type 2 diabetes mellitus a consensus algorithm for the initiation and adjustment of therapy: a consensus statement from the American Diabetes Association and the European Association for the Study of Diabetes. Diabetologia.

[2] Riddle M. C., Rosenstock J., Gerich J. (2003). Insulin Glargine Study I, The treat-to-target trial: randomized addition of glargine or human NPH insulin to oral therapy of type 2 diabetic patients. Diabetes Care.

[3] Rosenstock J., Ahmann A. J., Colon G. (2008). Advancing insulin therapy in type 2 diabetes previously treated with glargine plus oral agents: prandial premixed (insulin lispro protamine suspension/lispro) versus basal/bolus (glargine/lispro; therapy. Diabetes Care.

[4] Inzucchi S. E., Bergenstal R. M., Buse J. B. (2015). Management of hyperglycaemia in type 2 diabetes 2015: a patient-centered approach. Update to a position statement of the American Diabetes Association (ADA) and the European Association for the Study of Diabetes (EASD). Diabetes Care.

[5] Diamant M., Nauck M. A., Shaginian R. (2014). 4B Study Group, Glucagon-like peptide 1 receptor agonist or bolus insulin with optimized basal insulin in type 2 diabetes. Diabetes Care.

[6] Rosenstock J., Fonseca V. A., Gross J. L. (2014). Advancing basal insulin replacement in type 2 diabetes inadequately controlled with insulin glargine plus oral agents: a comparison of adding albiglutide, a weekly GLP-1 receptor agonist, versus thrice-daily prandial insulin lispro. Diabetes Care.

[7] Shao N., Kuang H. Y., Hao M. (2014). Effects of exenatide on obesity and non-alcoholic fatty liver disease with elevated liver enzymes in patients with type 2 diabetes. Diabetes Metab Res Rev.

[8] Raskin P., Bode B. W., Marks J. B. (2003). Continuous subcutaneous insulin infusion and multiple daily injection therapy are equally effective in type 2 diabetes. Diabetes Care.

[9] Herman W. H., Ilag L. L., Johnson S. L. (2005). A clinical trial of continuous subcutaneous insulin infusion versus multiple daily injections in older adults with type 2 diabetes. Diabetes Care.

[10] Berthe E., Lireux B., Coffin C. (2007). Effectiveness of intensive insulin therapy by multiple daily injections and continuous subcutaneous insulin infusion: a comparison study in type 2 diabetes with conventional insulin regimen failure. Horm Metab Res.

[11] Wainstein J., Metzger M., Boaz M. (2005). Insulin pump therapy versus multiple daily injections in obese type 2 diabetic patients. Diabet Med.

[12] Reznik Y., Morera J., Rod A. (2010). Efficacy of continuous subcutaneous insulin infusion (CSII) in type 2 diabetes mellitus: a survey on a cohort of 102 patients with prolonged follow-up. Diabetes Technol Therap.

[13] Charras L., Sanz C., Labrousse-Lhermine F. (2012). Traitement par pompe à insuline dans le diabète de type 2 (DT2): 12 ans de suivi d’une cohorte de 50 patients (Abstract). Diabetes Metab.

[14] Courrèges J. P., Donnet J. P., Gouet D. (2012). Résultats métaboliques obtenus à 2 ans sous pompe à insuline ambulatoire chez des diabétiques de type 2 en échec d’insulinothérapie optimisée (Abstract). Diabetes Metab.

[15] Frias J. P., Bode B. W., Bailey T. S. (2011). A 16-week open-label, multicenter pilot study assessing insulin pump therapy in patients with type 2 diabetes suboptimally controlled with multiple daily injections. J Diabetes Sci Technol.

[16] Kesavadev J., Balakrishnan S., Ahammed S., Jothydev S. (2009). Reduction of glycosylated haemoglobin following 6 months of continuous subcutaneous insulin infusion in an Indian population with type 2 diabetes. Diabetes Technol Ther.

[17] Reznik Y., Cohen O., Aronson R. (2014). OPT2MISE study group, Insulin pump treatment compared with multiple daily injections for treatment of type 2 diabetes (OpT2mise) a randomised open-label controlled trial. Lancet.

[18] Garvey W. T., Olefsky J. M., Griffin J. (1985). The effect of insulin treatment on insulin secretion and insulin action in type II diabetes mellitus. Diabetes.

[19] Gormley M. J., Hadden D. R., Woods R. (1986). One month’s insulin treatment of type II diabetes: the early and mediumterm effects following insulin withdrawal. Metabollsm.

[20] Pouwels M. J., Tack C. J., Hermust A. R., Lutterman J. A. (2003). Treatment with intravenous insulin followed by continuous subcutaneous insulin infusion improves glycaemic control in severely resistant type 2 diabetic patients. Diabet Med.

[21] Wainstein J., Metzger M., Wexler I. D. (2001). The use of continuous insulin delivery systems in severely insulin-resistant patients. Diabetes Care.

[22] Parkner T., Laursen T., Vestergaard E. T. (2008). Insulin and glucose profiles during continuous subcutaneous insulin infusion compared with injection of a long-acting insulin in type 2 diabetes. Diabet Med.

[23] Reznik Y., Morello R., Zenia A. (2014). Autonomy of patients with type 2 diabetes with an insulin pump device: is it predictable?. J Diabetes Sci Technol.

[24] Grunberger G., Abelseth J., Bailey T. (2014). Consensus statement by the American association of clinical endocrinologists/American college of endocrinology insulin pump task force. Endocr Pract.

[25] Reznik Y., Huang S. (2014). OpT2mise Study Group. Reductions in A1C with pump therapy in type 2 diabetes are independent of C-peptide and anti-glutamic acid decarboxylase antibody concentrations. Diabetes Technol Ther.

